# *RAB24* Missense Variant in Dogs with Cerebellar Ataxia

**DOI:** 10.3390/genes16080934

**Published:** 2025-08-04

**Authors:** Cleo Schwarz, Jan Wennemuth, Julien Guevar, Francesca Dörn, Vidhya Jagannathan, Tosso Leeb

**Affiliations:** 1 Institute of Genetics, Vetsuisse Faculty, University of Bern, 3001 Bern, Switzerland; cleo.schwarz@unibe.ch (C.S.); vidhya.jagannathan@unibe.ch (V.J.); 2 Graduate School for Cellular and Biomedical Sciences, University of Bern, 3012 Bern, Switzerland; 3 Department of Diagnostic Imaging, Veterinary Clinic Hofheim, 65719 Hofheim, Germany; j.wennemuth@tierklinik-hofheim.de; 4 Section of Clinical and Comparative Neuropathology, Centre for Clinical Veterinary Medicine, Ludwig Maximilians University, 80539 Munich, Germany; j.guevar@gmail.com; 5 Clinique Vétélys, 1214 Vernier, Switzerland; 6 Department of Neurology, Veterinary Clinic Kalbach, 60437 Frankfurt, Germany; francesca.doern@web.de

**Keywords:** *Canis lupus familiaris*, WGS, neurology, CNS, precision medicine, animal model

## Abstract

Hereditary ataxias are a highly heterogenous group of diseases characterized by loss of coordination. In this study, we investigated a family of random-bred dogs, in which two siblings were affected by a slowly progressive ataxia. They presented with clinical signs of progressive cerebellar ataxia, hypermetria, and absent menace response. The MRI revealed generalized brain atrophy, reduced cortical demarcation, hypoplastic corpus callosum, and cerebellar folia thinning, highly suggestive of a neurodegenerative disorder. We sequenced the genomes of the two affected dogs and their unaffected parents. Filtering for protein-changing variants that had homozygous alternate genotypes in the affected dogs, heterozygous genotypes in the parents, and homozygous reference genotypes in 1576 control genomes yielded a single missense variant in the *RAB24* gene, XM_038534663.1:c.239G>T or XP_038390591.1:p.(Gly80Val). Genotypes at this variant showed the expected co-segregation with the ataxia phenotype in the investigated family. The predicted amino acid affects the conserved RabF4 motif. Glycine-80 resides at the protein surface and the introduction of a hydrophobic isopropyl side chain of the mutant valine might impede solvent accessibility. Another missense variant in RAB24, p.Glu38Pro, was previously reported to cause a clinically similar form of cerebellar ataxia in Gordon Setters and Old English Sheepdogs. Taken together, the available data suggest that RAB24:p.Gly80Val represents the causal variant in the studied dogs. To the best of our knowledge, this is only the second report of a potentially pathogenic *RAB24* variant in any species and further supports that *RAB24* should be considered a candidate gene in human ataxia patients with unclear molecular etiology.

## 1. Introduction

Canine hereditary ataxias are a heterogeneous group of neurological diseases characterized by cerebellar or spinocerebellar dysfunction and resulting in uncoordinated movement as their hallmark [1]. Variants in more than 35 genes have been associated with canine ataxia. Based on the neuropathological alterations, five different subgroups have been proposed [1], which comprise cerebellar cortical degenerations [2,3,4,5,6], primary granule cell degenerations, spinocerebellar degenerations [7,8,9,10,11,12], cerebellar ataxia without substantial neurodegeneration [13,14,15], and multifocal degenerations with a predominant (spino)cerebellar component [16,17,18,19,20,21,22,23,24,25,26,27,28,29,30,31,32,33,34,35,36]. The ataxia may represent the only clinical sign or may be part of a syndromic disease. While most canine hereditary ataxias represent neurodegenerative disorders due to defects in the genes required for the metabolism and homeostasis of cerebellar neurons, ataxia can also result from impaired brain development [37]. The developmental ataxias may be characterized by clinical phenotypes that remain stable or even show improvements over time [37].

Due to the enormous heterogeneity of inherited ataxias, it is normally not possible to reach a precise diagnosis based on clinical signs alone. To aid in the diagnosis, advanced diagnostic imaging, metabolic analyses, pathological examinations, and/or genetic investigations are required.

The present study was prompted by reports of two dog littermates presenting with progressive loss of coordination and ataxia. The aim of our study was to characterize the clinical phenotype together with an investigation of the underlying causative genetic variant.

## 2. Materials and Methods

### 2.1. Ethics Statement

All of the dogs in this study were privately owned. Blood samples were collected with the consent of the owners. The diagnostic workup of the affected dogs was performed in the context of veterinary care and did not constitute an animal experiment in the legal sense. The collection of blood samples from control animals was approved by the Cantonal Committee for Animal Experiments (Canton of Bern; permit BE94/2022). All animal experiments were performed in accordance with local laws and regulations.

### 2.2. Index Family

The index family consisted of five dogs, two unaffected parents and three male offspring, of which two were affected by ataxia. The sire was reported as an American Bulldog and the dam as an Old English Bulldog, both without official pedigree papers. Samples and information on the five dogs were donated by their owners.

### 2.3. Clinical Examination and Diagnostic Imaging

The clinical and neurological examinations were performed by a veterinarian (neurologist in training, FD), and the MRI interpretation was performed by a board-certified specialist in diagnostic imaging (Dipl.-ECVDI, JW). The MRI images were taken using a Vet-MR Grande instrument with 0.25 Tesla field strength (Esaote Vet, Köln, Germany).

### 2.4. DNA Isolation and Whole-Genome Sequencing

Genomic DNA was isolated from the EDTA blood from all five dogs on a Maxwell RSC48 instrument using the Maxwell RSC Whole Blood DNA Kit (Promega, Dübendorf, Switzerland). PCR-free libraries with ~400 bp insert size were prepared from genomic DNA of the two affected dogs and their parents. The libraries were sequenced with 2 × 150 bp paired-end chemistry at 22×–27× coverage on an Illumina NovaSeq 6000 instrument (Illumina, San Diego, CA, USA). The raw reads were mapped to the UU_Cfam_GSD_1.0/CanFam4 genome reference assembly (GCF_011100685.1). Subsequently, single-nucleotide variants and small indels were indicated as described before [38]. The sequence data were deposited in the European Nucleotide Archive, and the accession numbers are listed in Table S1. The functional effects of the called variants were predicted with the SnpEff version 4.3t software [39], together with NCBI annotation release 106 for the UU_Cfam_GSD_1.0 genome reference assembly to produce an annotated vcf-file.

### 2.5. Variant Filtering

For the identification of candidate causal variants, we used a hard filtering approach on the annotated vcf-file with the following conditions: (1) genotypes in the two affected dogs had to be homozygous alternate (1/1), genotypes in the parents had to be heterozygous (0/1), and genotypes in 1576 control genomes from genetically diverse dogs had to be homozygous for the reference genotype (0/0). For the control genomes, we additionally allowed missing genotypes (./.). Accession numbers of the control genomes are listed in Table S1. In a second step, protein-changing variants were prioritized. We considered variants with a SnpEff predicted impact of “high” or “moderate” as protein-changing.

### 2.6. Targeted Genotyping

The *RAB24*:XM_038534663.1:c.239G>T candidate variant was independently confirmed and genotyped by direct Sanger sequencing of the PCR amplicons. Primers 5′-CCGGACGGTGACTTTAGGTA-3′ and 5′-CACGCAGAGAACAACCAAGA-3′ were used for the generation of a 393 bp amplicon containing the variant. PCR products were amplified from genomic DNA using AmpliTaq Gold 360 Master Mix (Thermo Fisher Scientific, Reinach, Switzerland). Direct Sanger sequencing of the PCR amplicons on an ABI 3730 DNA Analyzer (Thermo Fisher Scientific) was performed after treatment with exonuclease I and alkaline phosphatase. Sanger sequences were analyzed using the Sequencher 5.1 software (Gene Codes, Ann Arbor, MI, USA).

### 2.7. In Silico Pathogenicity Prediction and Structural Modeling

The online classification tools Polyphen-2 [40], PredictSNP [41], and MutPred2 [42] were utilized to predict the potential functional impact of the XP_038390591.1:p.Gly80Val missense variant. The Mol*3D viewer [43] from the RCSB Protein Data Bank was used to visualize the protein structure of the canine RAB24 protein (XP_038390591.1) as modeled with ColabFold [44].

## 3. Results

### 3.1. Clinical Phenotype

Two 5-month-old male random-bred (American Bulldog x Old English Bulldog) siblings presented with similar contemporaneous clinical signs of progressive gait abnormalities. Only case #1 was clinically examined in detail. The general clinical examination was within normal limits. The neurological examination showed normal consciousness and behavior. An examination of the cranial nerves identified an absent menace response bilaterally. The gait was abnormal with cerebellar ataxia with hypermetria. Intention tremor was also evident. Proprioception and spinal reflexes were within normal limits. Neuroanatomical localization was to the cerebellum, with differential diagnoses including degenerative, inflammatory, and hereditary conditions. Owner-provided videos of both cases illustrate the clinical phenotype (Video S1).

**Figure 1:**
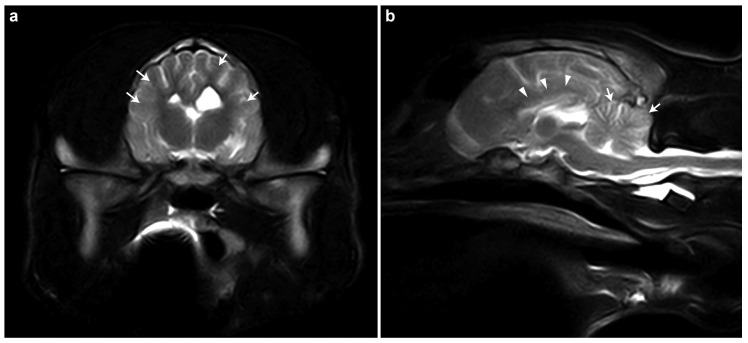
MRI findings in case #1. (**a**) T2-weighted transverse MRI image of the brain. Generalized reduction in the demarcation between the cortical gray matter and the subcortical white matter within the region of the corona radiata can be seen (white arrows). (**b**) T2-weighted sagittal MRI image of the brain. The corpus callosum is markedly thinned and poorly delineated (white arrowheads), suggesting hypoplasia. Additionally, the cerebellar folia are diffusely attenuated (white arrows), indicating cerebellar atrophy or hypoplasia.

The brain MRI of case #1 revealed generalized brain atrophy, reduced cortical demarcation, hypoplastic corpus callosum, and cerebellar folia thinning (Figure 1). These findings were primarily consistent with a neurodegenerative disorder, potentially a storage disease, with secondary generalized atrophy of the cerebrum and cerebellum. Inflammatory/infectious or metabolic disorders were judged to be less likely.

The complete blood test of case #1 was unremarkable. Cerebrospinal fluid (CSF) analysis revealed a cell-poor fluid (3 cells/µL, reference < 15) with no cytological evidence of inflammation, infection, or neoplastic cells. The biochemical parameters showed CSF total protein at 12 mg/dL (reference < 60 mg/dL), glucose at 80 mg/dL (reference 50−75 mg/dL), and lactate at 12.4 mg/dL (reference 11.0−19.0 mg/dL). Further specific tests for IgA, CrP, and TBE-Ab (IgG) in CSF, along with PCR for distemper, *Anaplasma phagocytophilum*, *Neospora caninum*, and *Toxoplasma gondii*, were all negative, supporting a non-inflammatory and non-infectious etiology for the neurological signs observed.

### 3.2. Genetic Analysis

We sequenced the genomes of both of the affected dogs and their parents and compared the sequence data to 1576 genetically diverse control genomes in a search for plausible causative variants. Assuming recessive inheritance, we searched for variants that had homozygous mutant (alternate) genotypes in the two affected dogs, heterozygous genotypes in both parents, and homozygous wildtype (reference) genotypes in the controls. A total of 13 variants in the entire genome fulfilled these search criteria, and only one of them was predicted to be protein-changing (Table 1 and Table S2).

**Figure 2:**
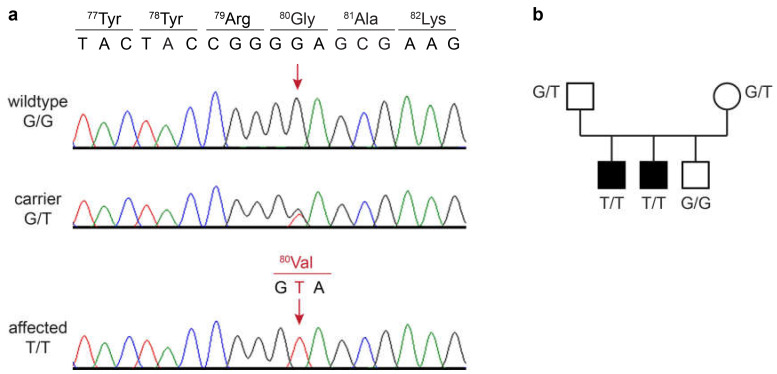
Details of the *RAB24*:c.239G>T variant. (**a**) Sanger electropherograms of a wildtype dog, a heterozygous carrier, and an affected dog. The position of the variant is shown with red arrows. The reading frame and amino acid translations of the wildtype and mutant sequence are indicated. (**b**) Genotype–phenotype co-segregation in the family. Affected dogs are represented with filled symbols. Genotypes at the *RAB24*:c.239G>T variant are indicated.

The identified single-candidate variant was located on chromosome 4, NC_049225.1:g.36,943,081G>T. It is a missense variant in the *RAB24* gene, XM_038534663.1:c.239G>T, predicted to change a glycine into a valine residue at the protein level, XP_038390591.1:p.(Gly80Val). The variant and correct co-segregation of the genotypes in the family were verified by Sanger sequencing. Both of the affected dogs were homozygous mutant, their parents were heterozygous, and the unaffected littermate was homozygous for the wildtype allele (Figure 2).

**Figure 3:**
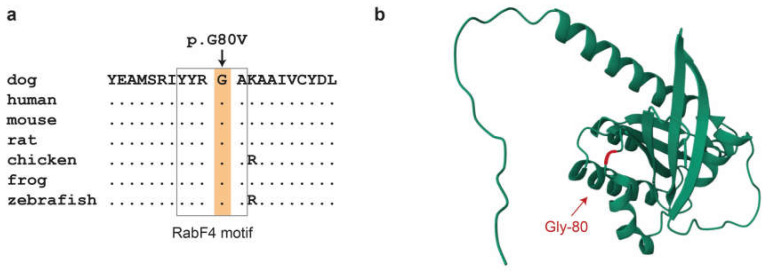
In silico analyses of the RAB24:p.Gly80Val variant. (**a**) Evolutionary conservation. The glycine at position 80 is strictly conserved in all vertebrates. Gly-80 is part of the RabF4 motif that is a conserved feature distinguishing Rab family members from other members of the Ras superfamily [45]. Accession numbers of the sequences used for the alignment are dog, XP_038390591.1; human, NP_001026847.1; mouse, NP_033026.1; rat, NP_001015023.2; chicken, NP_001264642.1; Western clawed frog, NP_001016171.1; zebrafish, NP_001025141.1. (**b**) Three-dimensional structure model of the canine RAB24 protein. Gly-80 is in a solvent exposed loop connecting the α2 helix with the β4 sheet.

The predicted Gly80Val amino acid substitution affects the RabF4 motif [45]. This is a highly conserved YYRGA sequence motif at the surface of the RAB24 protein (Figure 3). The mutant valine introduces a hydrophobic isopropyl side chain into the surrounding solvent, which might interfere with the normal contacts to water molecules.

In silico pathogenicity prediction tools classified the p.Gly80Val variant as potentially deleterious. MutPred2 gave a score of 0.890, which is above the 5% pathogenicity threshold of 0.8. PredictSNP classified the p.Gly80Val variant as deleterious with 87% probability. Polyphen-2 classified the variant as probably damaging with a score of 0.982.

## 4. Discussion

In this study, we investigated two dog siblings affected by hereditary ataxia. Using a powerful quartet whole-genome sequencing approach, we identified RAB24:p.Gly80Val as the candidate causal variant for this phenotype.

The constellation of clinical signs in young dogs (less than a year of age), particularly the cerebellar ataxia, hypermetria, intention tremor, and absent menace response, strongly pointed towards a cerebellar involvement. The MRI findings of generalized mild brain atrophy, reduced cortical demarcation, hypoplastic corpus callosum, thinning of cerebral gyri, and prominent sulci were highly suspicious of a metabolic or degenerative encephalopathy. A lysosomal storage disease with secondary generalized atrophy of both the cerebrum and cerebellum was suspected. Atrophy of the corpus callosum has been reported in animals with neuronal ceroid lipofuscinosis (NCL) and gangliosidosis [45], although it is not specific to these conditions and can also occur in other conditions [46].

The CSF analysis was overall unremarkable and suggestive of a non-inflammatory/infectious process. The mildly elevated glucose in CSF could be a minor variation or non-specific finding given the lack of other inflammatory markers.

Based on the clinical and MRI findings, a neurodegenerative process was the main differential diagnosis. Histopathology, typically the next step in confirming a diagnosis of neurodegenerative disorder, could not be performed as both dogs were still alive. According to the owners, the affected dogs had a good quality of life, albeit being coordinatively impaired. This underscores the importance of genetic testing in living patients for the investigation of neurodegenerative disorders. Based on the findings in Gordon Setters and Old English Sheepdogs with *RAB24*-related ataxia that have been studied for more than 40 years [4,47,48,49,50,51], the most likely course of disease in the affected dogs from this study will be slowly progressive.

The function of the RAB24 protein has only partially been clarified. It is highly conserved in all vertebrates and comprises 213 amino acids in dogs and humans, of which 211 are identical. RAB24 belongs to the Rab family within the Ras superfamily of small GTPases [52,53]. While most Ras proteins have a GTPase activity, RAB24 is considered an atypical Rab GTPase, as it lacks a conserved glutamine in the catalytic domain and therefore has only weak or absent GTPase activity and is mostly found in a GTP-bound state [53]. RAB24 is involved in intracellular transport and autophagy [54,55]. A more recent study reported a function in mitochondrial dynamics in the liver [56]. To the best of our knowledge, ataxic Gordon Setters and Old English Sheepdogs, homozygous for the mutant allele at RAB24:p.Gln38Pro, represent the only spontaneous *RAB24* mutant with a well-defined clinical phenotype [4]. So far, no human patients with *RAB24* loss of function variants have been reported.

The RAB24:p.Gly80Val variant detected in this study affects a highly conserved RabF4 sequence motif (YYRGA) [52]. The functional impact of this variant is predicted as deleterious or damaging by three different software tools. Based on three-dimensional structure modeling, it is conceivable that the introduction of the hydrophobic valine at the protein surface causes alterations in water solubility that might affect RAB24 function.

For a formal assessment of pathogenicity, we applied the ACMG/AMP guidelines for human variants [57]. We considered three criteria, one strong and two supporting, as evidence for pathogenicity: The homozygous mutant genotype was only seen in the two affected dogs, whereas 1576 genetically diverse dogs did not carry the mutant allele (PS4). The genotypes at RAB24:p.Gly80Val showed the expected co-segregation with disease in the family (PP1), and multiple lines of computational evidence supported pathogenicity (PP3). With these criteria, the variant would be classified as likely pathogenic.

While RAB24:p.Gly80Val represents a highly plausible candidate causal variant, we have to caution that our analyses had some limitations. We did not perform any functional confirmation experiments to prove that the mutant RAB24 is inactive. Furthermore, we relied on short-read sequencing data and only investigated small variants. We did not consider large structural variants. Gaps in the genome reference assembly and/or incorrect genome annotation might also have caused us to miss the true causal variant. Therefore, our assessment of RAB24:p.Gly80Val being likely causal should be considered preliminary and requires further confirmation before it can be taken as definitively proven. Functional studies on the effect of the p.Gly80Val substitution on the RAB24 protein are required to clarify the exact molecular pathogenesis. However, while we acknowledge the limitations of our study, the remarkable similarity in clinical phenotype between *RAB24* mutant ataxic Gordon Setters and Old English Sheepdogs [4] with the dogs investigated in our study strongly supports RAB24:p.Gly80Val as a candidate causal variant.

We found the mutant *RAB24* allele only in the index family consisting of non-pedigree dogs with owner-reported American Bulldog and Old English Bulldog origin, respectively. Further research is warranted to determine whether the mutant allele exclusively occurs in non-pedigree dogs or whether it is also segregating in purebred dogs.

## 5. Conclusions

We characterized the clinical phenotype of two dog littermates with an autosomal recessive hereditary ataxia. The genetic investigation identified *RAB24*:c.239G>T, a single-nucleotide variant resulting in the p.Gly80Val missense change as a most likely causative defect. To the best of our knowledge, this is only the second *RAB24* mutant with a neurological phenotype reported in any species. Therefore, our findings corroborate earlier findings on the previously reported other canine *RAB24* mutant. *RAB24* should be considered a candidate gene for unexplained forms of hereditary ataxia in human patients. Knowledge of the canine *RAB24* variant enables genetic testing to avoid the unintentional mating of carriers and production of further affected dogs.

## Figures and Tables

**Table 1:** Overview of variant filtering results. The genomes of the two affected littermates and their parents were analyzed together with 1576 control genomes. Private variants have the genotype 1/1 in case #1 or case #2 and the genotype 0/0 or ./. in the 1576 control genomes.

Filtering Step	Case #1	Case #2	Shared	Shared and Both Parents Heterozygous
Total homozygous variants in genome	2,953,576	2,866,164	2,131,515	74,890
Private homozygous variants	344	255	31	13
Private protein-changing homozygous variants	2	5	1	1

## Data Availability

The accession numbers of the sequence data that are reported in this study are listed in Table S1.

## Supplementary Materials

The following supporting information can be downloaded at: https://www.mdpi.com/article/10.3390/genes16080934/s1, Table S1: Accession numbers of 1580 dog genome sequences; Table S2: Private protein-changing variants in the two affected dogs; Video S1: Clinical presentation of the two affected dogs.
